# Comparison of Anatomical and Visual Outcomes between Idiopathic and Myopic Macular Holes Using the Internal Limiting Membrane or Inverted Internal Limiting Membrane Flap Technique

**DOI:** 10.1155/2019/6723824

**Published:** 2019-05-07

**Authors:** Federico Peralta Iturburu, Claudia Garcia-Arumi, Maria Bové Alvarez, Jose Garcia-Arumi

**Affiliations:** ^1^Instituto de Microcirugía Ocular (IMO), Barcelona, Spain; ^2^Hospital Universitario Vall d'Hebron, Barcelona, Spain

## Abstract

**Purpose:**

To compare the results of vitrectomy with those of internal limiting membrane (ILM) peeling or inverted ILM flap for treating myopic or idiopathic macular hole.

**Methods:**

Thirty-nine eyes of 39 patients undergoing vitrectomy with ILM peeling for macular hole (25 idiopathic and 14 myopic) and 27 eyes of 27 patients undergoing vitrectomy with inverted ILM flap (15 idiopathic and 12 myopic) were included. Outcome measures were macular hole closure by optical coherence tomography and visual acuity at 6 months.

**Results:**

Closure was achieved in 25 (100%) idiopathic and 12 (86%) myopic macular holes in the ILM peeling group and in 14 (93%) idiopathic and 11 (91.77%) macular holes in the inverted ILM flap group. There were no statistically significant differences in restoration of the external limiting membrane and ellipsoid zone between the groups. Median best-corrected visual acuity (logarithm of minimal angle of resolution) at the end of follow-up was 0.22 (20/32 Snellen) in idiopathic and 0.4 (20/50) in myopic (*P*=0.042) patients in the ILM peeling group and 0.4 (20/50) in idiopathic and 0.4 (20/50) in myopic (*P*=0.652) patients in the inverted ILM flap group.

**Conclusion:**

Both techniques were associated with high closure rates in myopic and idiopathic macular holes, with somewhat better visual outcomes in idiopathic cases. The small sample size may have provided insufficient power to support the superiority of one technique over the other in the two groups.

## 1. Introduction

The pathogenesis of macular hole (MH) is not fully understood and is considered to be multifactorial. It is believed to involve inwards traction from abnormal premacular vitreous cortex adhesion, thickening the internal limiting membrane (ILM) or epiretinal membrane, and in myopic MH, external traction from a posterior staphyloma [1, 2]. Surgery, a well-established treatment for MHs, has the aim of identifying and treating these vitreoretinal traction forces, which can be tangential, anteroposterior, or both.

Optical coherence tomography (OCT) has become a pivotal technique for the diagnosis and follow-up of idiopathic and myopic MHs, including the course of MH closure [3, 4]. The most common procedure for treating idiopathic MH is pars plana vitrectomy (PPV), posterior vitreous detachment, internal limiting membrane (ILM) peeling, filling of the vitreous cavity using a gas bubble, and postoperative face-down positioning [5]. In myopic MH, however, there is no widely accepted technique.

The inverted ILM flap technique, first described by Michalewska et al. [6], is a potentially useful surgical technique for treating large, full-thickness idiopathic MH and myopic MH. Although the anatomic closure rates are high with this procedure, there is no consensus as to whether the functional outcome is more favorable with the inverted ILM technique than with complete ILM peeling.

The purpose of this study was to evaluate the anatomic and functional outcomes in patients with idiopathic MH or myopic MH using the inverted ILM flap technique versus complete ILM peeling.

## 2. Methods

### 2.1. Study Design and Participants

This is a retrospective, nonrandomized, comparative study of consecutive patients with MH treated by a single vitreoretinal surgeon (J. G.-A.) using small-gauge PPV (23- or 25-gauge) at the Ocular Microsurgery Institute (IMO, Instituto de Microcirugía Ocular) in Barcelona, Spain, between January 2013 and January 2018. The study was approved by the IMO ethics committee and was conducted in accordance with the principles of the Declaration of Helsinki. Written informed consent for participation was obtained from all patients.

All patients underwent a complete ophthalmologic examination and spectral domain optical coherence tomography (SD-OCT) using the Cirrus HD-OCT 4000 or 5000 system (Carl Zeiss Meditec, Inc., Dublin, CA, USA) before surgery and at 1 week, 1 month, and 6 months after surgery. Minimum and maximum hole diameters were measured using OCT.

Patients were divided into subgroups according to the ILM removal technique (peeling vs. inverted flap) and axial length (AL) < 26 mm (idiopathic MH) or ≥26 mm (myopic MH). We included all eyes with a full-thickness MH without retinal detachment (RD) and follow-up of at least 6 months. The exclusion criteria were as follows: (1) traumatic MH or history of eye trauma, (2) macular edema of any cause, (3) recurrent MH, (4) presence of glaucoma or any other chronic ocular disease, and (5) presence of a systemic disease such as diabetes mellitus.

### 2.2. Surgical Procedure

Vitrectomies were performed under retrobulbar anesthesia. All patients underwent standard 3-port 23- or 25-gauge PPV with posterior vitreous detachment using a soft silicone-tipped cannula or active suction with the vitrectomy probe. Any epiretinal membrane present was peeled. The ILM was stained with a mixture of brilliant blue G and trypan blue (MembraneBlue-Dual; Dorc, Zuidland, the Netherlands) for 1 minute, which was then aspirated with a silicone-tipped cannula.

In the complete ILM peeling group, the ILM was peeled off in a circular manner for about two disk diameters around the MH using a forceps (Storz Single-Use Vitreoretinal Asymmetric Peeling Forceps, Aliso Viejo, CA) and then removed. The inverted ILM flap technique was performed according to the original description by Michalewska et al. [6] with some modifications. During ILM peeling around the MH, the ILM flap was not entirely removed from the retina; instead, a remnant was left attached to the MH periphery. To place the flap, a small amount of perfluorocarbon liquid was introduced over the macula. The remnant ILM flap was inverted with a silicone cannula and then pushed into the MH to fill it. The perfluorocarbon facilitated manipulation of the inverted flap and the other surgical maneuvers. In phakic patients, if lens opacification prevented visualization of the fundus, standard phacoemulsification with intraocular lens implantation was performed before vitrectomy. To avoid movement of the flap, the initial fluid-air exchange was performed peripherally to eliminate all fluid before removing the central perfluoro-*n*-octane (HPF8; Alchimia srl, Padova, Italy). Remnant fluid on the disk was then eliminated. After fluid-air exchange, we reconfirmed that the ILM flap remained in place. As the ILM flap was stained with brilliant blue G, it could be visualized through the air filling the vitreous cavity. Hence, gas tamponade (10% C3F8) was applied, and patients were instructed to maintain a prone position for approximately 5 days after surgery.

The complete postoperative ophthalmologic examinations included best-corrected visual acuity (BCVA), intraocular pressure, slit-lamp evaluation, fundus assessment, and SD-OCT. A high-resolution five lines scan was used to visualize the details of the fovea. MH closure was defined by SD-OCT as complete disappearance of the hole and absence of a neurosensory defect over the fovea. During each postoperative visit, the presence of photoreceptor (i.e., ellipsoid zone (EZ)) defects and external limiting membrane (ELM) was estimated. Recovered foveal microstructure layers were evaluated by standard 3 mm macular horizontal and vertical cross-sectional images with OTC. Restoration of ELM/ellipsoid layers was defined as continuous reflection lines. Flat-open and elevated-open myopic MHs were considered surgical failures.

### 2.3. Statistical Analyses

The BCVA was recorded as a decimal value and was converted to the logarithm of minimal angle of resolution (logMAR) and Snellen fraction for the statistical analysis. Fisher's exact test was used to compare categorical data. Student's *t*-test was used to compare normally distributed continuous data, and the nonparametric Mann–Whitney *U* test was applied when data were not normally distributed. Categorical data are expressed as the frequency and percentage and continuous data as the median and interquartile range (IQR) (25%–75%) when data were not normally distributed. Statistical significance for 2-sided tests was set at *P* < 0.05. Statistical analyses were performed using SPSS 15.0 (SPSS, Inc., Chicago, IL).

## 3. Results

Over the period of the study, 39 patients (18 men and 21 women) (39 eyes) underwent vitrectomy using the ILM peeling technique and 27 patients (15 men and 12 women) (28 eyes) were treated with vitrectomy using the inverted ILM flap procedure. The median (SD) age of the patients was 64.14 (±10.86) years, and the distribution of the study variables was similar in the two groups (Table 1). At baseline, there were no significant differences between idiopathic and myopic patients in the ILM peeling cohort (*P*=0.458) or in the ILM flap cohort (*P*=0.428) regarding MH diameter. Among myopic MH patients, 7 eyes in the ILM peeling group and 3 in the inverted flap group had an axial length >30 mm.

In patients treated with ILM peeling, average preoperative BCVA was 0.4 (IQR 0.7–0.3) logMAR (20/50 Snellen) in idiopathic MH and 0.6 (IQR 1–0.4) logMAR (20/80 Snellen) in myopic MH (*P*=0.119), whereas in the inverted ILM flap group, BCVA was 0.8 (1.3–0.6) logMAR (20/125 Snellen) in idiopathic and 0.45 (0.8–0.4) logMAR (20/56 Snellen) in myopic MH (*P* ≤ 0.001).

There were no statistical differences regarding the minimum diameter (*µ*m) of the MHs. Phacoemulsification in combination with PPV was performed in 8 eyes (21%) in the peeling group and in 3 (25%) in the inverted flap group.

Functional and anatomic results are shown in Table 2. In the peeling group, mean postoperative BCVA was 0.22 (IQR 0.3–0.1) logMAR (20/32 Snellen) in idiopathic MH and 0.4 (IQR 0.7–0.22) logMAR (20/50 Snellen) in myopic MH (*P*=0.042). In patients undergoing the inverted flap technique, BCVA was 0.4 (0.7–0.3) logMAR (20/50 Snellen) in idiopathic and 0.4 (0.85–0.3) logMAR (20/50 Snellen) in myopic MH (*P*=0.652).

In the group of <26 mm axial length, there was a statistical difference in the results of BCVA between ILM peeling vs. inverted flap group (*P* < 0.01). In the group of >26 mm axial length, there was not a statistical difference between ILM peeling vs. inverted flap (*P*=0.98).

**Figure 1:**
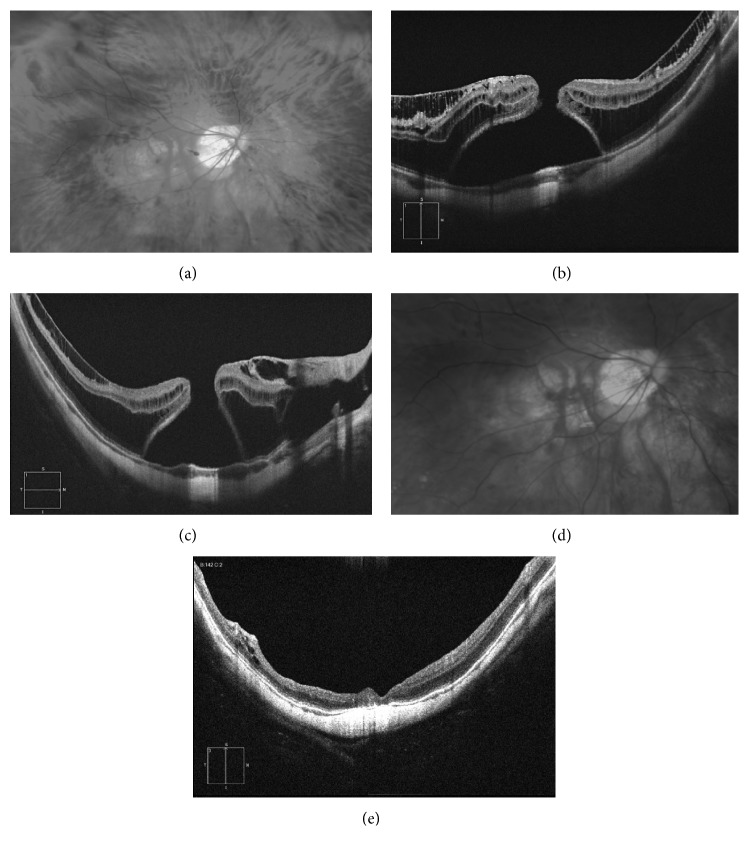
46-year-old woman with an axial length of 30.98 mm and a BCVA of 20/400, with inner and external retinoschisis and myopic macular hole. (a) Fundus photograph showing the chorioretinal atrophy and the macular hole. (b, c) Structural OCT showing the inner and external retinoschisis and the macular hole with subretinal fluid. Vitrectomy followed by inverted flap ILM technique was performed. (d) Postoperative retinography showing closure of the hole. (e) Structural OCT whit macular hole closure, disappearance of retinoschisis, but bad restoration of external retinal layers.

**Figure 2:**
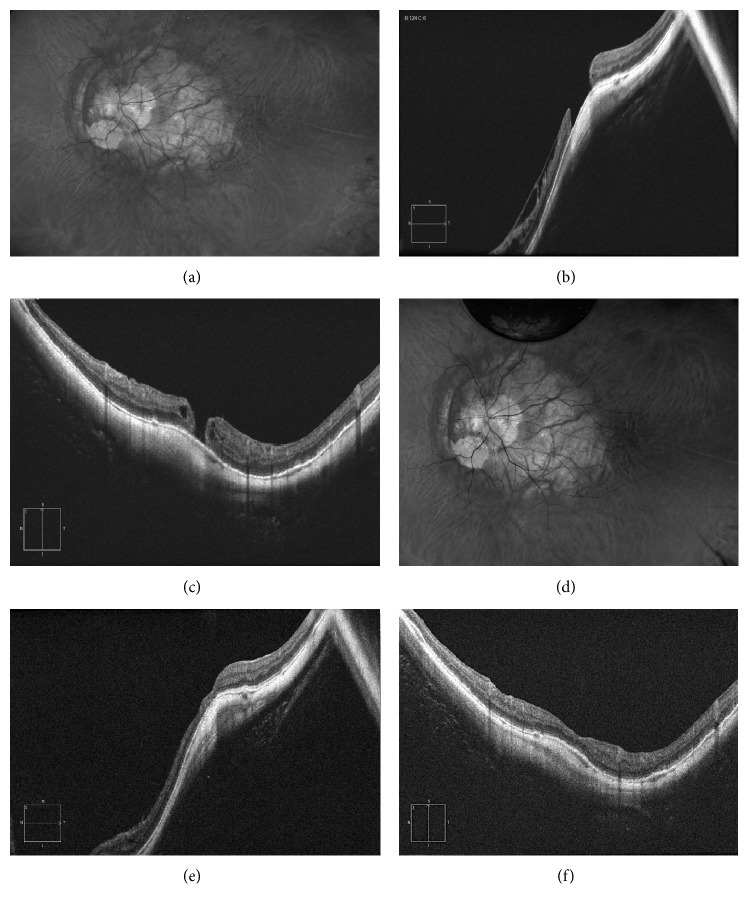
56-year-old man with an axial length of 38 mm and a BCVA of 20/400, with inner retinoschisis and a myopic macular hole. (a) Fundus retinography showing the posterior staphyloma involving macula and optic disc and the chorioretinal atrophy. (b, c) Structural horizontal and vertical OCT showing the inner retinoschisis and the macular hole of 650 microns. (d) Fundus retinography after vitrectomy and inverted flap technique. (e, f) Structural vertical and horizontal scan showing the closure of the macular hole, the resolution of the retinoschisis, and the restoration of all retinal layers.

Following vitrectomy with ILM peeling, MH closure was achieved in 100% (25 eyes) with AL < 26 mm and in 86% (12 eyes) with AL ≥ 26 mm (*P*=0.012). In patients undergoing vitrectomy with inverted ILM flap, 93% (14 eyes) with AL < 26 mm and 91.7% (11 eyes) with AL ≥ 26 mm (*P*=1) achieved MH closure (Figures 1 and 2). On SD-OCT at 6 months of follow-up in patients with closed MH, the external limiting membrane (ELM)/ellipsoid layers were restored in 19 of 25 eyes (AL < 26 mm) and in 5 of 12 eyes (AL ≥ 26 mm) in the ILM peeling group (*P*=0.168) and in 5 of 14 eyes (AL < 26 mm) and in 5 of 11 eyes (AL ≥ 26 mm) in the inverted ILM flap group (*P*=0.697). There were no statistically significant differences between any of the groups regarding restoration of the ELM and ellipsoid zone.

## 4. Discussion

The results of this study showed a similar closure rate with the use of the inverted ILM flap technique between eyes with AL < 26 mm (idiopathic MH) and AL ≥ 26 (myopic MH), although idiopathic MHs in the ILM peeling group had a somewhat higher closure rate (*P*=0.012).

The minor diameter of macular hole at the baseline in the ILM peeling group than in the inverted ILM flap group in the idiopathic macular hole (265 *μ*m respect of 465 *μ*m) can be the reason for the higher closure rate in the first group compared with the second one. It could also be explained, in our retrospective study, as ILM peeling technique was the preferred technique for the surgeon (G-A), in cases of macular hole <400 *µ*m.

Vitrectomy for MH, first described by Kelly and Wendel [7], has been the gold standard technique for this condition, although the success rates are much lower in large-diameter MH and in myopic eyes. ILM peeling and gas tamponade is a well-established surgical option for myopic MH without RD, and for cases without [8, 9] or with associated retinoschisis [10]. Closure rates after vitrectomy and ILM peeling are usually high in most studies. In the present series, closure rates were 100% (idiopathic) and 86% (myopic), values that are similar to the rates reported by others. In 24 highly myopic eyes with full-thickness MH without RD, García-Arumí et al. [3] reported successful anatomic MH closure at 6 months postoperatively in 87.5% of patients undergoing 1 surgery and in 100% with 2 surgeries. Other authors have reported closure rates of 83% to 100% [11]. With regard to the BCVA, the percentage of patients with a vision improvement after ILM peeling ranges from 41% to 83% [11, 12, 13, 14]. In the present study, BCVA increased in 76% of idiopathic MH and 71% of myopic MH cases (ILM peeling group), with no statistical differences.

In the original study of the inverted flap technique by Michalewska et al. [6], MH closure was achieved in 88% of patients undergoing standard 3-port PPV and in 98% of patients treated with the inverted ILM flap technique, in both large idiopathic MHs (>400 mm) and in myopic MHs. The authors hypothesized that this approach may induce glial cell proliferation, resulting in MH filling with proliferating cells that enhance closure as well as new positioning of photoreceptors in direct proximity to the fovea [6]. Other authors have also reported successful surgical repair of myopic MH using the inverted ILM flap [15, 16]. The present study, including patients with idiopathic MH and myopic MH, found that vitrectomy associated with standard ILM peeling was more effective in idiopathic than in myopic cases, whereas in the inverted flap group, there were no differences. In our patients undergoing ILM peeling, the closure rate was 100% in idiopathic MH and 86% in myopic MH, similar to the values reported in previous studies [17–19]. In the inverted ILM flap group, the closure rates of 93% in idiopathic and 91.7% in myopic MH were similar to those reported by Michalewska et al. [8, 15] in myopic MH. In the ILM peeling group, a BCVA improvement at the end of follow-up was observed in 76% of patients with AL < 26 mm//idiopathic and 71% of patients with AL ≥ 26 mm//myopic MH. In the inverted flap group, a BCVA improvement at the end of follow-up was documented in 87% of patients with AL < 26 mm//idiopathic and 50% of patients with AL ≥ 26 mm//myopic MH.

Restoration lines (ellipsoid zone and ELM) were evaluated by OCT. The better visual outcome in the idiopathic MH peeling group could be related to a higher percentage of recovery of both these layers at 6 months as compared to patients with myopic MH (76% vs. 42%). In the inverted flap group, the less favorable visual outcome may be related to the lower restoration rates in both idiopathic MH (36%) and myopic MH (20%). These findings suggest that the peeling technique may result in a greater BCVA improvement because of more complete restoration of the ELM and ellipsoid zones. It has been shown that ELM integrity is critical for achieving a normal IS/OS postoperatively [14, 20]. Wakabayashi et al. [20] have reported that evidence of ELM integrity on SD-OCT at 3 months is the most significant structural feature for predicting the 1-year postoperative BCVA in surgically closed MH.

The limitations of this study include a retrospective design, small sample size, and absence of randomization for the type of treatment. A larger sample size would likely have improved the statistical power of the study and more properly supported the conclusions. The degree of myopic chorioretinal atrophy is known to limit good visual outcome [21, 22], but this factor was not evaluated due to the difficulty of establishing a definite diagnosis. Nor was the relationship between restoration lines and visual acuity investigated. Finally, a follow-up substantially greater than 6 months would be needed to compare the long-term effects of the two techniques on improvement of visual acuity.

In summary, this study provides further evidence of high MH closure rates associated with use of either the ILM peeling technique or the inverted ILM flap technique in the management of patients with idiopathic or myopic MH. The results for visual outcome and restoration of the external layers were better with ILM peeling than with the inverted ILM flap procedure, but these findings should be interpreted with caution because of the relatively small sample and retrospective nature of the study. In this respect, conclusions regarding the superiority of one technique over the other cannot be established.

## Figures and Tables

**Table 1:** Preoperative characteristics of patients with MMH without RD undergoing ILM peeling or inverted ILM flap technique.

Variables	ILM peeling technique			Inverted ILM flap technique		
	AL < 26 mm (*n* = 25)	AL ≥ 26 mm (*n* = 14)	*P*	AL < 26 mm (*n* = 15)	AL ≥ 26 mm (*n* = 12)	*P*
Sex						
Men (*n* (%))	12 (48)	6 (43)	0.51	6 (40)	6 (50)	0.270
Women (*n* (%))	13 (52)	8 (57)		9 (60)	6 (50)	
Age (years, median (IQR))	66 (60.7–74.5)	63 (59.2–72.2)	0.458	65 (58.5–71.5)	59.5 (56–67)	0.428
Eye						
Right (*n* (%))	9 (36)	7 (50)	0.50	3 (20)	3 (25)	1.00
Left (*n* (%))	16 (64)	7 (50)		12 (80)	9 (75)	
AL (mm, median (IQR))	23.69 (23.40–23.83)	30.09 (27.21–31.63)	0.001	23.29 (22.86–23.41)	28.7 (25.8–30)	0.001
AL >30 (%)		7 (50)			3 (25)	
Refractive error (diopters, median (IQR))	−1 (−2 to 0.25)	−13.19 (−18 to −8.62)	<0.001	−1.25 (−2 to −0.12)	−9.5 (−13.8 to −7.3)	0.001
Posterior staphyloma (%)	0	11 (79)		0	5 (42)	
Lens status						
Phakic	3 (12)	0	0.540	1 (7)	5 (42)	0.060
Phakic intraocular lens	22 (88)	14 (100)		14 (93)	7 (58)	
BCVA (logMAR, median (IQR))	0.4 (0.7–0.3)	0.6 (1–0.4)	0.119	0.8 (1.3–0.6)	0.45 (0.8–0.4)	0.001
BCVA (Snellen chart, median (IQR))	20/50 (20/100–20/40)	20/80 (20/200–20/50)		20/125 (20/400–20/80)	20/56 (20/126–20/50)	
Macular hole						
Minimum diameter (*µ*m, median (IQR))	267 (125–324)	414 (294–559)	0.218	496 (362–633)	307 (170–453)	0.020
Chronic MH (*n* (%))	7 (28)	1 (7)	0.218	4 (27)	5 (45)	0.448

AL, axial length; BCVA, best-corrected visual acuity; ILM, internal limiting membrane; MH, macular hole.

**Table 2:** Anatomical and functional results in patients undergoing ILM peeling or inverted ILM flap technique in relationship with the axial length: AL < 26 mm or AL ≥ 26 mm.

Variables	ILM peeling technique			Inverted ILM flap technique		
	AL < 26 mm (*n* = 25)	AL ≥ 26 mm (*n* = 14)	*P*	AL < 26 mm (*n* = 15)	AL ≥ 26 mm (*n* = 12)	*P*
Macular hole						
Open	0	2 (14)	0.012	1 (7)	1 (8.3)	1.000
Closed at 6 months	25 (100)	12 (86)		14 (93)	11 (91.7)	
Chronic MH						
Closed	7/7 (100)	0/1	—	3/4 (75)	4/5 (80)	1.000
Restoration of ELM/ellipsoid zone at 6 months	19/25 (76)	5/12 (42)	0.168	5/14 (36)	5/11 (45)	0.697
BCVA (logMAR), median (IQR)						
At 6 months	0.22 (0.3–0.1)	0.4 (0.7–0.22)	0.042	0.4 (0.7–0.3)	0.4 (0.85–0.3)	0.652
BCVA (Snellen chart), median (IQR)						
At 6 months	20/32 (20/40–20/25)	20/50 (20/100–20/32)	0.138	20/50 (20/100–20/40)	20/50 (20/141–20/39)	0.652
Increased BCVA						
At 6 months	19 (76)	10 (71)	0.142	13 (87)	6 (50)	0.031
Increased or stable BCVA						
At 6 months	25 (100)	12 (86)	0.123	15 (100)	9 (75)	0.075

AL, axial length; BCVA, best-corrected visual acuity; ELM, external limiting membrane; ILM, internal limiting membrane; MH, macular hole.

## Data Availability

The data used to support the findings of this study are available from the corresponding author upon request.
